# Adult hippocampal neuroplasticity triggers susceptibility to recurrent depression

**DOI:** 10.1038/tp.2017.29

**Published:** 2017-03-14

**Authors:** N D Alves, J S Correia, P Patrício, A Mateus-Pinheiro, A R Machado-Santos, E Loureiro-Campos, M Morais, J M Bessa, N Sousa, L Pinto

**Affiliations:** 1 Life and Health Sciences Research Institute (ICVS), School of Medicine, University of Minho, Braga, Portugal; 2 ICVS/3B's - PT Government Associate Laboratory, Braga/Guimarães, Portugal

## Abstract

Depression is a highly prevalent and recurrent neuropsychiatric disorder associated with alterations in emotional and cognitive domains. Neuroplastic phenomena are increasingly considered central to the etiopathogenesis of and recovery from depression. Nevertheless, a high number of remitted patients experience recurrent episodes of depression, remaining unclear how previous episodes impact on behavior and neuroplasticity and/or whether modulation of neuroplasticity is important to prevent recurrent depression. Through re-exposure to an unpredictable chronic mild stress protocol in rats, we observed the re-appearance of emotional and cognitive deficits. Furthermore, treatment with the antidepressants fluoxetine and imipramine was effective to promote sustained reversion of a depressive-like phenotype; however, their differential impact on adult hippocampal neuroplasticity triggered a distinct response to stress re-exposure: while imipramine re-established hippocampal neurogenesis and neuronal dendritic arborization contributing to resilience to recurrent depressive-like behavior, stress re-exposure in fluoxetine-treated animals resulted in an overproduction of adult-born neurons along with neuronal atrophy of granule neurons, accounting for an increased susceptibility to recurrent behavioral changes typical of depression. Strikingly, cell proliferation arrest compromised the behavior resilience induced by imipramine and buffered the susceptibility to recurrent behavioral changes promoted by fluoxetine. This study shows that previous exposure to a depressive-like episode impacts on the behavioral and neuroanatomical changes triggered by subsequent re-exposure to similar experimental conditions and reveals that the proper control of adult hippocampal neuroplasticity triggered by antidepressants is essential to counteract recurrent depressive-like episodes.

## Introduction

Major depression is a prevalent neuropsychiatric disorder affecting around 16% of the population worldwide, which experiences one or several episodes of depression during their lifetime.^1^ Despite the moderate capacity to achieve remission, over 85% of remitted patients suffer recurrent episodes of depression, within 15 years after an initial event.^2, 3^ Although a first episode has been mostly linked to stressful events,^4^ recurrent depression has also been associated to the persistence of subclinical residual symptoms and the number of previous episodes.^5^ Nevertheless, these evidences rely on the risk to develop recurrence and not on the determinants of recurrence or relapse events. In this context, it is of the upmost importance to understand the biological mechanisms underlying the precipitation of recurrent episodes and determine the behavioral traits prone to re-appear.

Over the years, few efforts have been made to study the impact of repeated stress exposure. Recently, the deleterious effects evoked by re-exposure to stress were associated to altered expression of cytoskeletal proteins.^6^ Furthermore, other studies revealed that animals subjected to stress during adolescence were resilient to depressive- and anxiety-like behavior on chronic stress re-exposure in adulthood^7^ and abrogation of hippocampal neurogenesis in adolescence blocked susceptibility to chronic social defeat in adulthood.^8^

Still, the neuroplastic capacity of the adult brain, observed in regions such as the hippocampus, was not yet associated to resilience or susceptibility to recurrent depression. In this brain region, highly sensitive to the detrimental effects of stress, neuroplastic events, including adult cytogenesis (neurogenesis^9, 10, 11^ and gliogenesis^12^) and morphological changes, are described to be altered by chronic stress and reverted by antidepressants (ADs).^13, 14^ Neuroplastic processes, and particularly its disturbances, have behavioral repercussions in cognitive and emotional dimensions in both physiological^15^ and pathological conditions including schizophrenia^16^ and depressive disorders.^17, 18, 19^ Also, previous studies indicate that, at short-term, behavioral improvements induced by ADs rely on hippocampal neuronal remodeling,^9^ while at long-term, remission is mainly associated to a normalized production of newborn neurons in the adult hippocampal dentate gyrus (DG).^20^

Therefore, it is crucial to understand the impact of recurrent stress in behavior and neuroplastic processes and the importance of modulating neuroplasticity in the prevention of recurrent episodes of depression. As such, in this work, we assessed behavioral alterations induced by recurrent stress and the impact of typical ADs, fluoxetine and imipramine, by repeated exposure to unpredictable chronic mild stress (uCMS) protocol. Also, to evaluate the relevance of hippocampal cytogenesis in the effects of ADs, a subset of animals was simultaneously treated with methylazoxymethanol (MAM), a cytostatic agent that artificially blocks cell proliferation.^9, 20, 21, 22^ We observed that pre-treatment with fluoxetine or imipramine confers a different profile of response to stress re-exposure, which relies on their distinct impact in adult hippocampal neuroplasticity.

## Materials and methods

A brief description of the materials and methods is presented in this section. For a full description, please refer to the Supplementary Information.

### Animals

Experiments were conducted in adult male (2 months old) Wistar Han rats (Charles River Laboratories, L'Arbresle, France) housed and kept under standard laboratory conditions at 22±1 ºC, 55% relative humidity, 12 h light/dark cycle, food and water *ad libitum*. Rats (*n=*6–8 for behavioral analysis, of which three to four were considered for gene expression quantification, immunofluorescence and neuromorphologic studies) from two independent sets were randomly divided into six groups, next described in detail. All the procedures were conducted in accordance with EU Directive 2010/63/EU and the Portuguese National Authority for animal experimentation, *Direção-Geral de Alimentação e Veterinária*.

### uCMS and drug treatments

Rats were subjected to a validated uCMS protocol for 6 weeks, as previously described^9, 20, 23^ and detailed in the Supplementary Information. Similarly to previous studies,^9, 20^ in the last 2 weeks, animals were daily injected intraperitoneally with saline (SAL) or with ADs to induce behavioral recovery either with fluoxetine (FLX; 10 mg kg^−1^, Kemprotec, Middlesbrough, UK) or imipramine (IMIP; 10 mg kg^−1^; Kemprotec). Subgroups of animals, treated with ADs were co-administered with MAM (7 mg kg^−1^; MRI Global Chemical Carcinogen Repository, Kansas City, MO, USA; subcutaneously). Simultaneously, a group of animals not exposed to uCMS, were also injected with saline (CTRL). All groups received during 7 days (4 days before and 3 days after the cessation of the first uCMS protocol) intraperitoneal injections of bromodeoxyuridine (BrdU, 50 mg kg^−1^; Sigma-Aldrich, St. Louis, MO, USA) to label newly adult-born cells generated immediately after ADs treatment. For the following 4 weeks after uCMS, animals were not subjected to any stressor. Afterwards, animals were re-exposed to a slight modified version of the uCMS protocol, detailed in the Supplementary Information, to avoid habituation to stressors.

### Behavioral analysis

**Figure 1:**
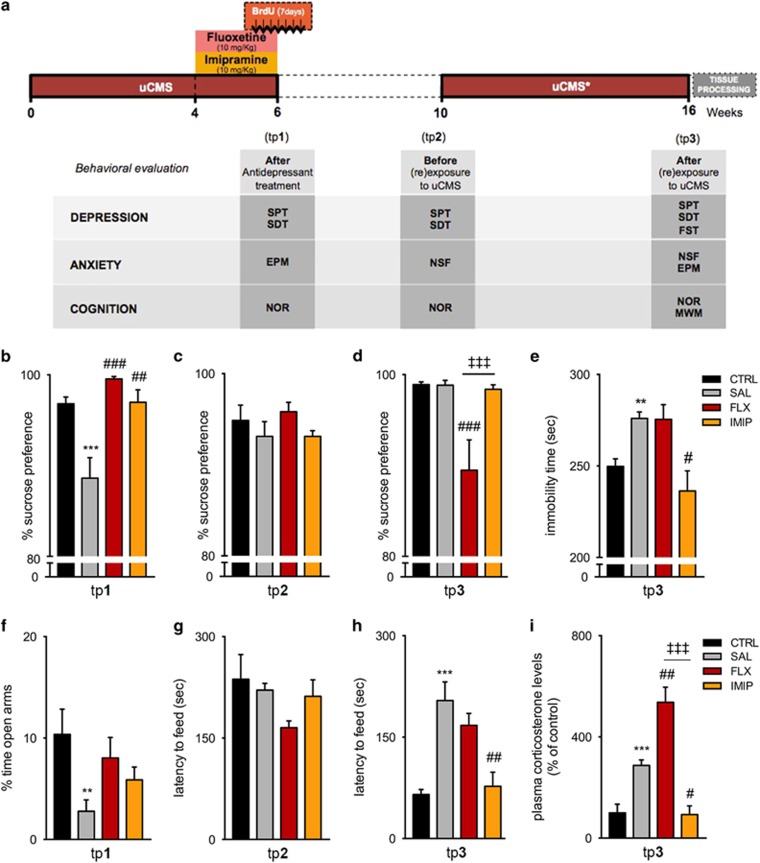
Anhedonic- and anxiety-like behaviors induced by recurrent stress exposure are prevented by imipramine treatment, but not fluoxetine. (**a**) Schematic representation of the experimental timeline used, including behavioral assessments throughout the protocol and the respective treatments. (**b**–**d**) Longitudinal assessment of anhedonic-like behavior by the SPT, revealed an increased susceptibility after re-exposure to stress driven by fluoxetine. (**e**) Assessment of behavioral despair, at the end of the experimental protocol, by the FST test revealed that recurrent stress exposure induced a significant increase of immobility time, only prevented in animals treated with imipramine. (**f**–**h**) Anxiety-like behavior was continuously tested throughout the experimental protocol, at week 6 by the EPM (**f**) and the NSF at weeks 10 (**g**) and 16 (**h**), evidencing the efficacy of imipramine in preventing anxiety-like behavior after stress re-exposure, contrarily to fluoxetine. (**i**) Non-treated and particularly, fluoxetine-treated animals, subjected to repeated uCMS exposure presented elevated corticosterone levels in the serum. Basal corticosterone levels were measured in the serum of rats collected between 0800 and 0900 at the end of the protocol. See also Supplementary Figures 1 and 2. *Denotes the effect of uCMS analyzed by Student's *t*-test; ^#^Denotes the effect of ADs, by comparison of treatment and SAL animals; and ^‡^denotes differences between ADs, analyzed by one-way analysis of variance (ANOVA). Data are represented as mean±s.e.m. ^#^*P*⩽0.05, ^**, ##^*P*⩽0.01, ^***, ###, ‡‡‡^*P*⩽0.001; *n*=6–8 animals per group. uCMS, unpredictable chronic mild stress protocol (uCMS*, slightly modified version. See Supplementary Information). AD, antidepressant; CTRL, non-stressed animals; EPM, elevated-plus maze; FLX, animals repeatedly exposed to uCMS and treated with fluoxetine; FST, forced-swimming test; IMIP, animals repeatedly exposed to uCMS and treated with imipramine; MWM, Morris water maze; NOR, novel object recognition; NSF, novelty-suppressed feeding; SAL, animals repeatedly exposed to uCMS and non-treated; SDT, sweet-drive test; SPT, sucrose preference test; TP, time point.

Along the experimental protocol, behavior was continuously monitored for depressive- and anhedonic-like behavior, anxiety and cognition (Figure 1a). At weeks 6, 10 and 16, animals were submitted to the sucrose preference test and sweet-drive test to assess anhedonic-like behavior and the novel object recognition for cognition. Anxiety-like behavior was assessed at week 6 through the elevated-plus maze and at weeks 10 and 16 by the novelty-suppressed feeding. At the end of the protocol, additional tests were performed: forced-swimming test for depressive-like behavior and the Morris water maze for cognition.

### Quantification of hippocampal newborn cells survival and neuronal morphometric analysis

Conditions and antibodies used in immunostaining for neurogenesis analysis was performed as described in detailed in Supplementary Information. Dendritic morphology of granule neurons from the dorsal DG was assessed by the Golgi-Cox method. Dendritic length and neuronal branching were analyzed as detailed in Supplementary Information.

### RT-PCR measurements

The mRNA expression levels of neuroplasticity-related genes were measured by qRT-PCR, as previously described.^24^ Total RNA was isolated from macrodissected DG using the Direct-zol RNA MiniPrep (Zymo Research, Irvine, CA, USA) according to the manufacturer's instructions. Obtained RNA (500 ng) was reverse transcribed using qScript cDNA SuperMix (Quanta Biosciences, Gaithersburg, MA, USA). Beta-2-Microglobulin (B2M) was used as internal standard for normalization of the target gene's expression. Genes analyzed and respective sequences are detailed in Supplementary Information. Real-time reactions were performed in Applied Biosystems 7500 Fast-Real Time PCR System (Applied Biosystems, Foster City, CA, USA) using 5 × HOT FIREPol EvaGreen qPCR Mix Plus (ROX) (Solis Biodyne, Tartu, Estonia). Relative gene expression was calculated using the ΔΔCt method. The results are presented as relative expression to the standard gene.

### Statistical analysis

Statistical analysis was performed using GraphPad Prism 6.0 (GraphPad Software, La Jolla, CA, USA). The animals were randomly assigned in experimental groups. The sample sizes were based on previous published studies.^9, 20^ All presented data satisfied normal distribution in Kolmogorov–Smirnov testing. After confirmation of homogeneity of group variances between the groups, data were subjected to appropriate statistical tests. Student's *t*-test was used for statistical comparisons between two groups when appropriate. The comparison between stressed groups was evaluated using one-way analysis of variance. Analysis of variance repeated measures was used to analyze cognitive learning tasks performance and Sholl analysis. Descriptive statistical results are presented as mean±s.e.m. Differences between groups were determined by Bonferroni's *post hoc* multiple comparison test and statistical significance was set at *P<*0.05. No data points were excluded from the different analyses.

## Results

### Non-treated and fluoxetine-treated animals, but not imipramine-treated animals, are behaviorally susceptible to recurrent uCMS exposure

To assess the impact of recurrent uCMS exposure and the relevance of ADs treatment, behavior domains typically affected by stress were monitored throughout the protocol (time points (tp) 1–3; Figure 1a), including depressive-like behavior, anxiety and cognition. As previously shown,^9, 25^ exposure to uCMS elicited an anhedonic state manifested by decreased sucrose preference in the sucrose preference test that was reverted by both fluoxetine and imipramine treatment (tp1, *P*=0.0008; F_2,14_=15.21, *P*=0.0003; FLX: *post hoc P*=0.0003; IMIP: *post hoc P*=0.0033, Figure 1b). This effect was preserved after a 4-week stress-free period (tp2). Spontaneous recovery of anhedonic behavior was also achieved in uCMS non-treated animals (*P*>0.1, Figure 1c). Strikingly and contrarily to other studies,^6^ uCMS re-exposure to non-treated animals did not promote the re-appearance of anhedonic-like behavior (tp3: *P*>0.1, Figure 1d); the same resilience was observed in animals treated with imipramine during the first uCMS exposure. However, and in accordance to previous reports,^6^ treatment with fluoxetine conferred susceptibility to anhedonic-like behavior as denoted by the significantly decreased sucrose preference (F_2,14_=16.11, *P*=0.0002; FLX: *post hoc P*=0.0003; IMIP: *post hoc P*>0.1, Figure 1d). These alterations in the anhedonic-like profile were corroborated by the recently developed sweet-drive test^26^ (Supplementary Figures 1a and b). Nevertheless, immobility time in the forced-swimming test, a typical surrogate measure of depressive-like behavior, was significantly increased after repeated uCMS exposure (Figure 1e), suggesting the re-appearance of depressive-like behavior in non-treated animals (*P*<0.01), similarly to those treated with fluoxetine (F_2,14_=7.078, *P*=0.0075; FLX: *post hoc P*>0.1; Figure 1e). In contrast, the treatment with imipramine successfully prevented the re-appearance of depressive-like behavior after uCMS re-exposure (IMIP: *post hoc P*=0.0112, Figure 1e).

**Figure 2:**
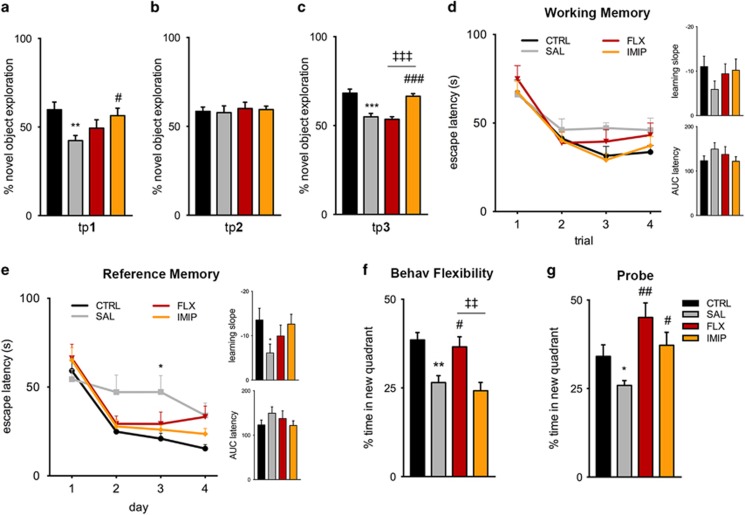
Evaluation of cognitive function throughout the experimental protocol revealed that imipramine is able to prevent cognitive deficits induced by recurrent stress exposure while fluoxetine specifically prevents alterations in behavior flexibility. (**a**–**c**) Continuous assessment of long-term memory using the novel object recognition (NOR) test revealed that recurrent stress induces cognitive deficits, which are also observed in fluoxetine-treated animals but not with imipramine treatment. (**d**–**g**) At the end of the protocol, the MWM test was used to evaluate cognitive performances, including working (**d**) and reference memory (**e**), reversal learning (**f**) and working memory by a probe trial (**g**). *Denotes the effect of uCMS analyzed by Student's *t*-test; ^#^Denotes the effect of ADs, by comparison of treatment and SAL animals; and ^‡^denotes differences between ADs analyzed by one-way analysis of variance (ANOVA). ANOVA repeated measures was used to analyze cognitive learning tasks performance. Data are represented as mean±s.e.m. ^*, #^*P*⩽0.05, ^**, ##, ‡‡^*P*⩽0.01, ^***, ###, ‡‡‡^*P*⩽0.001; *n*=6–8 animals *per* group. AD, antidepressant; AUC, area under the curve; CTRL, non-stressed animals; FLX, animals repeatedly exposed to uCMS and treated with fluoxetine; IMIP, animals repeatedly exposed to uCMS and treated with imipramine; MWM, Morris water maze; NOR, novel object recognition; SAL, animals repeatedly exposed to uCMS and non-treated; TP, time point; uCMS, unpredictable chronic mild stress protocol.

When tested for anxiety traits, uCMS exposure increased anxiety-like behavior in the elevated-plus maze as previously described,^9, 20^ which was partially rescued by both ADs (tp1: *P*=0.0071; F_2,14_=3.264, *P*=0.0686; FLX: *post hoc P*=0.0981; IMIP: *post hoc P*>0.1, Figure 1f). Following a 4-week stress-free period, no signs of anxiety-like behavior were observed in the novelty-suppressed feeding test in both non-treated and treated animals (tp2, Figure 1g). However, uCMS re-exposure promoted the re-emergence of an anxious-like phenotype in non-treated (tp3: *P*<0.001) but also in fluoxetine-treated animals (F_2,12_=8.786, *P*=0.0045; FLX: *post hoc P*>0.1). In contrast, imipramine-treated animals did not display signs of anxiety-like behavior after uCMS re-exposure (IMIP: *post hoc P*=0.0048, Figure 1h). No changes in food consumption were observed among groups (Supplementary Figures 2a and b). The analysis of plasma corticosterone levels in blood serum, after uCMS re-exposure, revealed elevated levels in non-treated (*P*<0.001) and fluoxetine-treated animals (F_2,9_=26.40, *P*=0.0002; FLX: *post hoc P*=0.0040, Figure 1i), when compared with non-stressed animals, whereas imipramine-treated animals presented corticosterone levels similar to the non-stressed group (IMIP: *post hoc P*=0.0288, Figure 1i). In addition, as stress dysregulates cognitive functions that depend on the structural integrity of the hippocampus, prefrontal cortex and reciprocal connections between these two regions, cognitive performance was also assessed (Figure 2). We observed that cognition was significantly affected by uCMS exposure, as it decreased novel object exploration in the novel object recognition test (tp1, *P*=0.0011, Figure 2a); notably, such impairment in long-term memory was significantly reversed by imipramine (tp1, F_2,36_=3.566, *P*=0.0386; FLX: *post hoc P*=0.6358; IMIP: *post hoc P*=0.0340, Figure 2a). Four weeks after cessation of the uCMS protocol, no cognitive deficits were observed in any experimental group (tp2, Figure 2b). However, uCMS re-exposure elicited the re-appearance of long-term memory deficits by decreasing novel object exploration in non-treated and fluoxetine-treated animals, but not imipramine-treated animals (tp3, *P*=0.0001; F_2,29_=1.482, *P*<0.0001; FLX: *post hoc P*>0.99; IMIP: *post hoc P*=0.0001, Figure 2c). Moreover, and despite no alterations in spatial working memory in the Morris water maze test (Figure 2d), uCMS re-exposure induced deficits in the reference memory task, particularly evident in the third day of the test (Figure 2e). In concordance, significant differences were observed for learning slope (*P*=0.0164) and area under the curve latency (*P*=0.0267; Figure 2e). In this specific task, treatment with fluoxetine or imipramine prevented such cognitive deficits (Figure 2e). Interestingly, impairments in spatial behavioral flexibility promoted by uCMS re-exposure in non-treated animals (*P*=0.0018, Figure 2f) were only prevented by fluoxetine, but not by imipramine (F_2,13_=7.521, *P*=0.0068; FLX: *post hoc P*=0.0264; IMIP: *post hoc P*>0.1, Figure 2f). Analysis of the probe trial for learning flexibility assessment also evidenced the impairments induced by repeated uCMS exposure (*P*=0.0257, Figure 2g). Prior treatment with fluoxetine or imipramine blocked the emergence of cognitive deficits on the probe trial (F_2,13_=9.241, *P*=0.0032; FLX: *post hoc P*=0.0032; IMIP: *post hoc P*=0.0484, Figure 2g).

### Stress re-exposure differently affects the generation of DG newborn neurons by ADs

**Figure 3:**
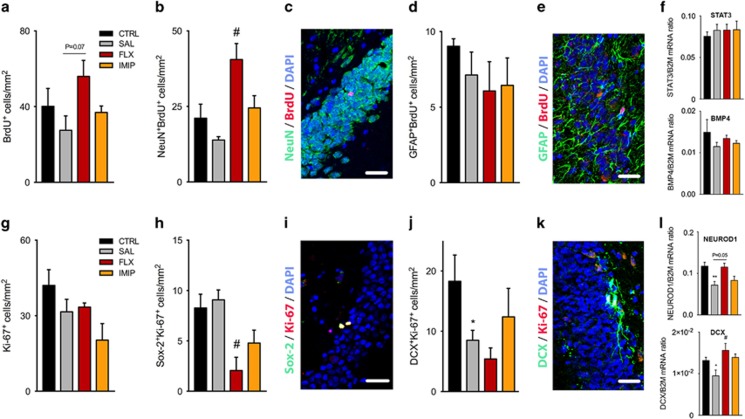
Treatment with fluoxetine boosts the generation and survival of newborn hippocampal neurons even after recurrent exposure to stress. (**a** and **b**) Quantification of the number of BrdU^+^ and BrdU^+^NeuN^+^ cells, per dentate gyrus (DG) area, revealed an increased production and survival of newborn neurons evoked by fluoxetine treatment. (**c**) Representative coronal section of the DG stained for BrdU (in red), NeuN (in green) and DAPI (in blue). (**d**) Density of newborn astroglial cells, identified as BrdU^+^GFAP^+^ cells, revealed no major effect of stress or ADs treatment. (**e**) Representative staining for BrdU (in red), GFAP (in green) and DAPI (in blue) in hippocampal DG. (**f**) Analysis of relative expression levels of STAT3 (upper panel) and BMP4 (lower panel) in the macrodissected DG corroborated the absence of major effects on gliogenesis in consequence of the recurrent stress exposure or AD treatment. (**g** and **h**) Quantitative analysis of Ki-67^+^ cells and amplifying progenitors, identified as Sox-2^+^Ki-67^+^ cells, revealed that fluoxetine leads to a depletion of DG progenitor cells. (**i**) Representative confocal image of Sox-2 (green) and Ki-67 (red) immunostaining. (**j**) Analysis of the number of DCX^+^Ki-67^+^ cells, representative of the neuroblasts population in the DG showed that animals subjected to repeated stress exposure, non-treated and treated with fluoxetine, presented a decreased number of neuroblasts. (**k**) Coronal section of the DG stained for DCX (in green), Ki-67 (in red) and DAPI (in blue). (**l**) Relative mRNA expression levels of NEUROD1 (upper panel) and DCX (lower panel) in the macrodissected DG revealed a decrease in the expression levels as a consequence of repeated stress exposure. In general, treatment with ADs restored the expression levels of both makers of neuronal maturation. Scale bars represent 30 μm. *Denotes the effect of unpredictable chronic mild stress (uCMS) analyzed by Student's *t*-test; ^#^denotes the effect of ADs, by comparison of treatment and SAL animals, analyzed by one-way analysis of variance (ANOVA). Data represented as mean±s.e.m. ^*, #^*P*⩽0.05; *n*=± 4 animals *per* group. AD, antidepressant; CTRL, non-stressed animals; FLX, animals repeatedly exposed to uCMS and treated with fluoxetine; IMIP, animals repeatedly exposed to uCMS and treated with imipramine; SAL, animals repeatedly exposed to uCMS and non-treated.

The impact of recurrent uCMS exposure and AD treatment in the process of adult neurogenesis was next assessed through the quantification of cell proliferation and newborn cells survival. No statistical differences were observed in the hippocampal cell proliferation or newborn cells in non-treated uCMS re-exposed animals, as well as in the imipramine-treated animals, in comparison with nonstressed animals (BrdU^+^: *P*=0.1792, F_2,6_=4.435, *P*=0.0657; IMIP: *post hoc P*>0.1, Figure 3a; BrdU^+^NeuN^+^: *P*=0.1584, F_2,5_=8.381, *P*=0.0253; IMIP: *post hoc P*>0.1, Figures 3b and c). On the other hand, the treatment with fluoxetine tends to increase the number of BrdU^+^ cells (FLX: *post hoc P*=0.0795; Figure 3a) and induced a significant increase of newborn neurons in the adult DG, identified as BrdU^+^NeuN^+^ cells (FLX: *post hoc P*=0.0319; Figures 3b and c). Interestingly, this effect was neuronal-specific, as no alterations were found in the number of newborn astroglial cells (identified as BrdU^+^GFAP^+^ cells; Figures 3d and e) and in the expression analysis of astroglial differentiation promoting factors, STAT3 and BMP4 (Figure 3f). In addition, quantification of the number of progenitor cells revealed no alterations in the number of Ki-67^+^ proliferating cells among groups (Figure 3g), but a significant decrease in the number of Ki-67^+^Sox-2^+^ progenitor cells in fluoxetine-treated animals was observed (F_2,7_=9.642, *P*=0.0097; FLX: *post hoc P*=0.0106, Figures 3h and i). Moreover, recurrent uCMS exposure produced a significant decrease in the number of neuroblasts, revealed by a decreased number of Ki-67^+^DCX^+^ (*P*=0.0186, Figures 3j and k), and also evidenced by the decreased gene expression of NEUROD1 and DCX (NEUROD1: *P*=0.0088; DCX: *P*=0.0481, Figure 3l). Fluoxetine-treated animals also presented a marked decrease in the number of neuroblasts, contrarily to those treated with imipramine (Figure 3j). Surprisingly, fluoxetine treatment tends to revert the decreased expression of NEUROD1 and DCX induced by recurrent stress (NEUROD1: F_2,6_=5.759, *P*=0.0402; FLX: *post hoc P*=0.0509; DCX: F_2,8_=5.170, *P*=0,0362; FLX: *post hoc P*=0.0419, Figure 3l).

### Effect of recurrent stress on neuronal morphology depends on AD treatment

**Figure 4:**
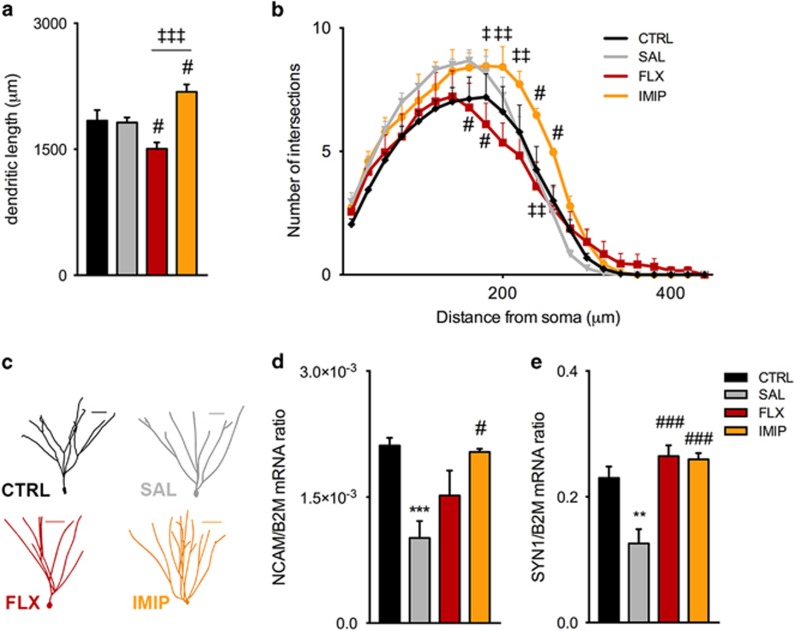
Imipramine enhances dentate gyrus (DG) neuronal arborization, whereas fluoxetine promotes an atrophy of granule neurons after recurrent stress. Dendritic length analysis (**a**) and neuronal organization (**b**) of DG granule neurons showed a dendritic shrinkage promoted by fluoxetine treatment, whereas imipramine induced an enlargement of the neuronal arborization after stress re-exposure. (**c**) Representative three-dimensional (3D) morphometric reconstruction of DG granule neurons of each experimental group. (**d** and **e**) The relative gene expression levels of remodeling genes, NCAM and SYN1, corroborates the neuronal remodeling promoted by imipramine treatment. Scale bars represent 50 μm. *Denotes the effect of unpredictable chronic mild stress (uCMS) analyzed by Student's *t*-test. ^#^Denotes the effect of ADs, by comparison of treatment and SAL animals; and ^‡^denotes differences between ADs, analyzed by one-way analysis of variance (ANOVA). ANOVA repeated measures was used to analyze Sholl analysis. Data are represented as mean±s.e.m. ^#, ‡^*P*⩽0.05, ^**, ‡‡^*P*⩽0.01, ^***, ###, ‡‡‡^*P*⩽0.001; *n*=± 4 animals per group. AD, antidepressant; CTRL, non-stressed animals; FLX, animals repeatedly exposed to uCMS and treated with fluoxetine; IMIP, animals repeatedly exposed to uCMS and treated with imipramine; SAL, animals repeatedly exposed to uCMS and non-treated.

In addition to hippocampal neurogenesis, changes in dendritic remodeling and synaptic plasticity of granule neurons were also assessed. Three-dimensional morphological analysis revealed that, although stress re-exposure in non-treated animals did not affect neuronal arborization, animals pre-treated with fluoxetine presented significant dendritic atrophy (Figures 4a and c). Conversely, imipramine-treated animals, on stress re-exposure presented a robust increase in neuronal dendritic arborization (F_2,8_=19.70, *P*=0.0008; FLX: *post hoc P*=0.0429; IMIP: *post hoc P*=0.0286, Figures 4a and c). In accordance, Sholl analysis revealed a reduced complexity of granule neurons in fluoxetine-treated animals, whereas imipramine promoted an overall increase in dendritic arborization (Figure 4b). Even though no major effect of stress re-exposure on neuronal atrophy was observed, gene expression analysis of neuroplastic-related genes revealed a marked decrease of NCAM (*P*=0.0002) and SYN1 (*P*=0.0027) expression, suggesting deficits in synaptic plasticity. Of notice, fluoxetine treatment was able to prevent changes in the expression of SYN1 and imipramine prevented alterations in the expression of both NCAM and SYN1 (NCAM: F_2,13_=4.890, *P*=0.0261; FLX: *post hoc P*=0.3926; IMIP: *post hoc P*=0.0240; SYN1: F_2,14_=22.64, *P*<0.001; FLX: *post hoc P*=0.001; IMIP: *post hoc P*<0.001, Figures 4d and e).

### Proliferation arrest blocks the behavioral effects of ADs after uCMS re-exposure

**Figure 5:**
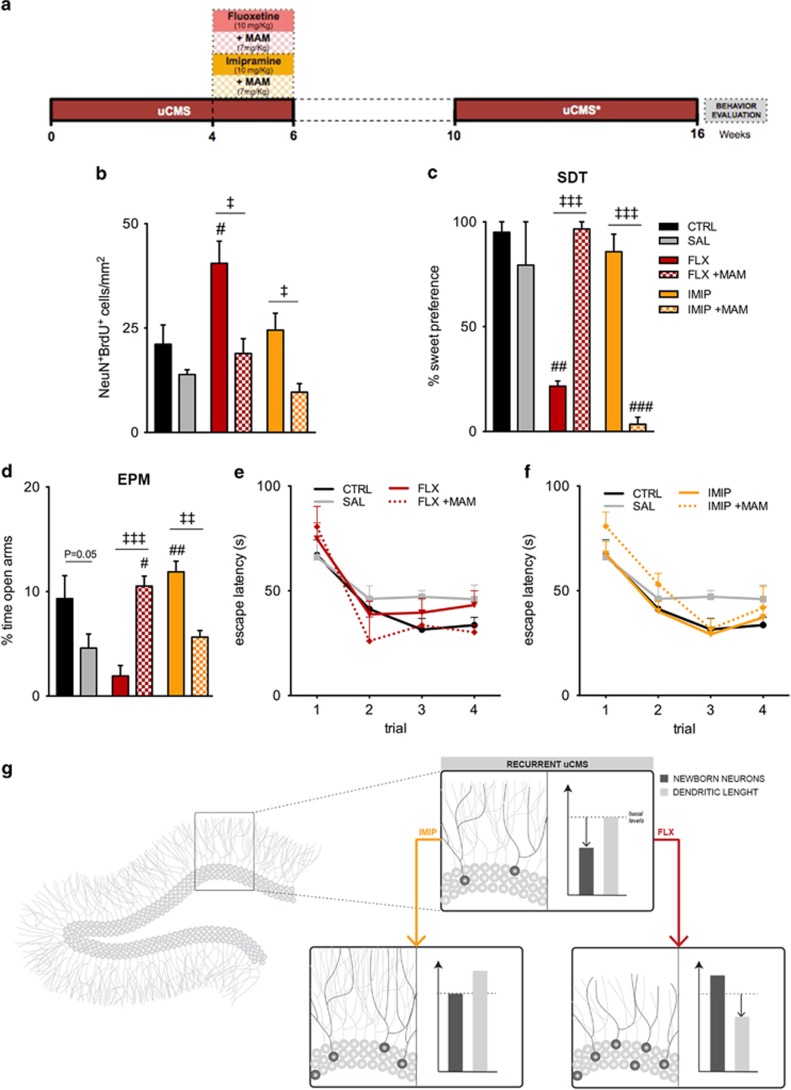
Ablation of adult hippocampal neurogenesis by MAM rescues behavioral deficits induced by fluoxetine in recurrent stress while inhibiting the protective effects promoted by imipramine. (**a**) Representative scheme of the experimental timeline used, including drug treatments with ADs and MAM. (**b**) Quantification of the number of BrdU^+^NeuN^+^ cells in the hippocampal dentate gyrus (DG), representing the population of newborn neurons denotes that proliferation arrest promotes a normalization of adult neurogenesis in consequence of treatment with fluoxetine, whereas a depletion in the levels of neurogenesis in animals treated with imipramine occurred. (**c**) The assessment of anhedonic-like behavior by the SDT, at the end of the protocol, revealed that the arrestment of adult neurogenesis rescues the anhedonic phenotype presented by fluoxetine-treated animals and inhibits the effect of imipramine to prevent anhedonic alterations. (**d**) Anxiety-like behavior, tested by the EPM, demonstrated that ablation of adult hippocampal neurogenesis has a preventive effect on anxiety-induced fluoxetine while leading to an anxious-like state when MAM was co-treated with imipramine. (**e** and **f**) MWM performed to assess cognitive performance on working memory revealed no major effects of MAM treatment in this behavioral dimension. (**g**) Schematic representation of the impact of adult neuroplasticity alterations promoted by unpredictable chronic mild stress (uCMS) and ADs. ^#^Denotes the effect of ADs, by comparison of treatment and SAL animals and ^‡^denotes differences between ADs, analyzed by one-way analysis of variance (ANOVA). ANOVA repeated measures was used to analyze cognitive learning tasks performance. Data are represented as mean±s.e.m. ^#, ‡^*P*⩽0.05, ^##, ‡‡^*P*⩽0.01, ^###, ‡‡‡^*P*⩽0.001; *n*=6–8 animals per group. AD, antidepressant; CTRL, non-stressed animals; EPM, elevated-plus maze; FLX, animals repeatedly exposed to uCMS and treated with fluoxetine; FLX+MAM, animals repeatedly exposed to uCMS and treated with fluoxetine and methylazoxymethanol; IMIP, animals repeatedly exposed to uCMS and treated with imipramine; IMIP+MAM, animals repeatedly exposed to uCMS and treated with imipramine and methylazoxymethanol; MAM, methylazoxymethanol; MWM, Morris water maze; SAL, animals repeatedly exposed to uCMS and non-treated; SDT, sweet-drive test.

To examine the requirement of adult hippocampal neurogenesis to mediate behavioral outcomes promoted by ADs in recurrent depression, a subgroup of ADs-treated animals was co-administered with MAM, for cell proliferation blockage, during the last 2 weeks of the first uCMS exposure (Figure 5a). Of notice, as previously observed,^20, 22, 27^ cell proliferation arrestment by MAM impacts anxiety-like behavior of non-stressed animals (Supplementary Figure 3). As expected, MAM administration in animals re-exposed to uCMS and co-treated with fluoxetine induced a significant reduction in the number of BrdU^+^NeuN^+^ cells, preventing the increased neurogenesis promoted by fluoxetine (FLX *vs* FLX+MAM: *P*=0.0303, Figure 5b). Concerning imipramine, co-treatment with MAM elicited significantly lower levels of neurogenesis, similar to those observed in stressed non-treated animals (IMIP vs IMIP+MAM: *P*=0.0153, Figure 5b).

Further assessment of anhedonic-like behavior by the sweet-drive test after uCMS re-exposure revealed that neurogenesis arrestment prevented anhedonic-like signs observed in animals co-treated with fluoxetine during the first uCMS exposure (FLX+MAM: *post hoc P*>0.1, Figure 5c). Remarkably, cell proliferation arrest led to the appearance of anhedonic-like behavior in animals co-treated with imipramine (IMIP+MAM: *post hoc P*<0.001, Figure 5c). In addition, the MAM treatment prevented anxiety-like signs promoted by fluoxetine (F_2,22_=15.70, *P*<0.001; FLX: *post hoc P*>0.1; FLX+MAM: *post hoc P*=0.0134, Figure 5d) while it compromised the therapeutic protection conferred by imipramine for this behavioral dimension, leading to an anxious-like state (IMIP: *post hoc P*=0.017; IMIP+MAM: *post hoc P*>0.1, Figure 5d).

Despite the importance of cell genesis modulation for the AD effects on emotional behaviors, results of the Morris water maze revealed that cell proliferation blockage by MAM had no significant effect on the cognitive performance of animals treated either with fluoxetine or imipramine (Figures 5e and f).

## Discussion

In the present work, using a well-established animal model of depression, we studied how re-exposure to chronic stress impacts in emotional, anxiety and cognitive behaviors. Noticeably, we showed that recurrent uCMS exposure potentiated the re-appearance of depressive- and anxiety-like behaviors as well as cognitive deficits. In accordance to previous reports, we observed that treatment with fluoxetine and imipramine promoted a sustained remission of an initial depressive-like state and a re-establishment of the hippocampal neurogenic process.^20^ However, uCMS induces persistent morphological, namely synaptic, and behavioral scars that are observed even 4 weeks post stress exposure. These residual changes, together with reported impairments in DG synaptic plasticity^9, 28, 29^ may account for the re-appearance of depressive, anxiety and cognitive deficits on uCMS re-exposure. Strikingly, our results also show that the impact of an initial uCMS, followed by a recovery period, increases animals' ability to cope with uCMS re-exposure in particular behavioral traits, such as anhedonia. These animals present intact DG dendritic length and no alterations in the survival of newborn neurons, which may account for the observed resilience to anhedonic-like deficits. However, the contribution of other brain regions, such as the nucleus accumbens, cannot be excluded of this anhedonic resilience, given the previous association between stress-induced anhedonia and medium spiny neurons hypertrophy at the nucleus accumbens.^25^

We next studied how treatment with typical ADs, fluoxetine and imipramine, during the first depressive-like episode, would impact in further recurrent episodes and, in addition, understand the importance of pro-neuroplastic effects triggered by ADs to prevent recurrent depression. Results revealed that animals treated with fluoxetine during the initial uCMS exposure presented an increased susceptibility to anhedonic-like behavior once re-exposed to uCMS, in contrast to non-treated animals, which did not present an anhedonic phenotype. Moreover, treatment with fluoxetine was inefficient in preventing the detrimental behavioral consequences induced by stress re-exposure, including depressive- and anxiety-like behavior as well as cognitive deficits (long-term memory) with the exception of a specific cognitive task (behavioral flexibility). Interestingly, these results seem to be in accordance with animal studies reporting behavioral deficits in fluoxetine-treated animals re-exposed to stress^6^ and in agreement with clinical studies showing that remitted patients treated with fluoxetine present higher rates of relapse, in comparison with other ADs.^30, 31^ Interestingly, co-administration of MAM and fluoxetine prevented the re-emergence of anhedonic- and anxiety-like behavior triggered by uCMS re-exposure. This observation suggests that the abnormal potentiation of cell proliferation and sustained increase in the number of adult-born neurons promoted by fluoxetine, previously described,^9, 20^ is most likely triggering the increased susceptibility to anhedonic-like behavior and contributing to the depressive- and anxiety-like phenotype induced by uCMS re-exposure (Figure 5g). In fact, an aberrant increase in the generation of newborn neurons was shown to enhance plasticity and excitability,^32^ increase the competition for afferent inputs^33^ and depolarization in response to GABA-ergic inputs.^34^ Consequently, such alterations promote an increased participation of these cells during learning and memory tasks^35^ and a reconfiguration of DG-CA3 circuits that degrade stored memories.^36^ Moreover, previous studies have shown an increased anxiety-like phenotype in consequence of increased neurogenesis induced by voluntary wheel running.^37, 38, 39^ In this context, our findings reinforce the view that re-establishment of adult hippocampal neurogenesis is required for a sustained remission from depressive-like behavior^20^ and that production and survival of adult-born neurons beyond a certain threshold can be detrimental for normal hippocampal function. Importantly, previous studies also suggested a strong impact of hippocampal structural alterations in the susceptibility to depression^40^ and an important contribution for recurrent episodes.^41^ Noticeably, in consequence of the higher recruitment of cells for differentiation caused by fluoxetine treatment, we also observed, as expected, a strong depletion in the number of progenitor cells and neuroblasts in the hippocampal DG. Despite the attempt to potentiate the neurogenic process, denoted by the increased expression of neuronal maturation-related markers, fluoxetine treatment was not able to restore the levels of neuronal differentiation possibly due to the depletion of neural progenitors. Besides boosting adult neurogenesis, fluoxetine treatment and further stress re-exposure also induced dendritic atrophy of the DG granule neurons, similar to the immediate effects induced by uCMS protocol in non-treated animals.^9^ Altogether, the non-physiological potentiation of neurogenesis and the neuronal atrophy promoted by fluoxetine may trigger increased susceptibility to behavioral deficits in stress recurrence, as shown here. Strikingly, fluoxetine-treated animals where resilient to a specific cognitive task, namely spatial reversal learning, that was compromised in non-treated animals. This particular cognitive task is not exclusively associated with hippocampal function but also the prefrontal cortex,^42^ namely the prelimbic and infralimbic, and orbitalfrontal regions;^43^ thus, there is the possibility that fluoxetine treatment may positively affect these prefrontal regions accounting to the observed resilience of reversal learning deficits. Contrastingly, treatment with imipramine conferred protection to uCMS re-exposure in the majority of behavioral domains assessed, including cognitive, and anxiety-like behavior. This behavioral resilience to repeated uCMS was associated with a normalization of the hippocampal neurogenic process,^20^ but also with a robust increase in the dendritic arborization of DG granule neurons (Figure 5g). In fact, the maintenance of adult neurogenesis at certain levels was required to prevent behavior deficits induced by recurrent stress, as the suppression of cell genesis by co-administration of MAM prevented the resilience to behavioral alterations on treatment with imipramine. In addition, and in line with previous studies demonstrating that ADs, including imipramine, reverse the shrinkage in dendritic branching induced by stress and allows the re-establishment of the normal neuronal network,^9^ we here suggest that the observed long-term increase in the arborization of DG granule neurons also accounts for the increased resistance to repeated stressful events. Accordingly, it has been proposed that adaptation to stress driven by neuronal structural alterations is a source of resilience that, when absent, contributes to the onset and recurrence of neuropsychiatric disorders, such as depression.^44, 45^ Likewise, imipramine is also known to confer protection to depressive-like behavior through an increase in the dendritic arborization of immature neurons.^46^ It is plausible that the distinct effects promoted by fluoxetine and imipramine, in case of re-exposure to stress, could be linked to their differential impact on neural cell types^14^ and/or to their distinct selectivity to neuronal receptors. The latter hypothesis could be explored in future studies through the specific blockage of the noradrenergic component on treatment with imipramine.

This study shows that a recurrent stress exposure promotes the re-appearance of anxiety and cognitive deficits. Moreover, these behavioral alterations were correlated with normal levels of survival of newborn neurons with exception of synaptic plasticity that showed persistent impairments after recurrent stress exposure. Our findings put forward the understanding how previous stressful events may have short-term detrimental effects on cortico-limbic plasticity but also highlight their importance in conferring protection against certain behavioral and neuroplastic deficits after recurrent stressful situations. In addition, the present work is the first to show that abnormal adult neuroplasticity triggers distinct responses to recurrent exposure to uCMS: an exaggerated potentiation of adult neurogenesis and neuronal atrophy increases the susceptibility to specific behavioral deficits induced by recurrent stress exposure, whereas normalization of adult neurogenesis, and an enhancement of dendritic arborization, confers protection to recurrent depression. In essence, these findings reflect how adult neuroplasticity changes induced by stress and ADs must be finely tuned to prevent recurrence in depression.
