# Supplementary material for: Adult hippocampal neuroplasticity triggers susceptibility to recurrent depression
Source: Transl Psychiatry. 2017 Mar 14;7(3):e1058–. doi: 10.1038/tp.2017.29 (PMC5416672; doi:10.1038/tp.2017.29)
Supplement: Supplementary Methods and Figures [file tp201729x1.docx]

**Adult hippocampal neuroplasticity triggers susceptibility to recurrent depression**

**Running title:** Neuroplasticity sets recurrence in depression

**Alves ND^1^**^,2^, Correia JS^1,2^, Patrício P^1,2^, Mateus-Pinheiro A^1,2^, Machado-Santos AR^1,2^, Loureiro-Campos E^1,2^, Morais M^1,2^, Bessa JM^1,2^, Sousa N^1,2^, Pinto L^1,2*^

**Supplementary Information**

**Supplementary Methods**

**Unpredictable chronic mild stress (uCMS)**

A validated uCMS protocol previously described[^1-3^](#_ENREF_1) was applied for periods of 6 weeks. Briefly, this stress paradigm induces depressive-like behavior as well as anxiety-like phenotype and cognitive deficits in rats through random and unpredictable exposure to a wide range of different mild stressors. The initial protocol included distinct stressors as confinement to a restricted space for 1 h, placement in a tilted cage (30º) for 3 h, housing on damp bedding for 8 h, exposure to noise during 4 h, overnight illumination, 15 h of food deprivation followed by exposure to inaccessible food for 1 h, water deprivation for 15 h followed by exposure to an empty bottle for 1 h, exposure to stroboscopic lights during 4 h, and reversed light/dark cycle for 48 h every 7 days. To increase unpredictability and avoid habituation to stressors, the subsequent uCMS protocol included additional stressors, namely removal of sawdust for 3 h, replacement of sawdust with cold water (4ºC) for 2 h and the switch of animals’ mates for 2 h.

**Behavioral analysis**

*Sucrose preference test (SPT)*

Anhedonia was assessed by the SPT, as previously described[^4^](#_ENREF_4), at several timepoints throughout the experimental protocol. Before each trial, rats were food and water deprived for 12 h. For testing, two pre-weighed bottles containing water or a 2% (m/v) sucrose solution were presented to individually housed animals for one hour. Sucrose preference (SP) was calculated according to the formula: SP = (sucrose intake / (sucrose intake + water intake)) x 100. Baseline SP value was assessed immediately before the start of the uCMS protocol. Anhedonia was defined as a reduction in SP relative to baseline levels.

*Sweet drive test (SDT)*

The SDT test was used as an additional continuous measure of anhedonic-like behavior, as previously described[^5^](#_ENREF_5). Briefly, animals food deprived for 10 h were placed in the SDT apparatus to freely explore during 10 min, with the SDT arena containing pre-weighted sweet pellets (Cheerios®, Nestlé, Vevey, Switzerland) in the right chamber and pre-weighted regular pellets (4RF21-GLP; Mucedola, Settimo Milanese, Italy) in the left arena. Three SDT trials were conducted (one trial every 48 h). Preference for sweet pellets was defined as a measure of anhedonic-like behavior and calculated as follows: Preference for sweet pellets (%) = Consumption of Sweet Pellets (g) / Total Food Consumption (g) x 100.

*Novelty-suppressed feeding (NSF)*

Anxiety-like behavior was assessed though the NSF test as previously described[^4^](#_ENREF_4), before (tp2) and after (tp3) the second exposure to the uCMS protocol, in two independent groups of animals. After 18 h of food-deprivation, animals were placed for a maximum of 10 min in an open-field arena containing a single food pellet in the center. The latency to feed was used as an index of anxiety-like behavior. After reaching the pellet, animals were returned to the home cage and allowed to feed during 10 min. Food consumption provided a measure of appetite drive (**Supplementary Figure 2**).

*Elevated-plus maze (EPM)*

To examine anxiety-like behavior, the EPM test was also used[^6^](#_ENREF_6). The behavioral apparatus (ENV-560; Med Associates Inc., St. Albans, VT, USA) consisted of two opposite open arms (50.8 cm × 10.2 cm) and two closed arms (50.8 cm × 10.2 cm × 40.6 cm) elevated 72.4 cm above the floor and dimly illuminated. Animals were individually placed in the center of the maze and allowed to freely explore it during 5 min. Each trial was video-recorded and the percentage of time spent in the open arms was measured using EthoVision XT 11.5 tracking system (Ethovision, Noldus Information Technologies, Wageningen, The Netherlands) as an index of anxiety-like behavior.

*Forced swimming test (FST)*

Behavioral despair was assessed through the FST, as previously described[^7^](#_ENREF_7). Rats were individually placed in transparent cylinders (62 cm height and 25.4 cm diameter) filled with water (25ºC; 50 cm depth) for 5 min. Testing was conducted 24 h after a 5-min pretest session. Trials were video-recorded and the total time animals spent immobile (immobility time) was measured using the EthoVision XT 11.5 tracking system (Noldus Information Technology). Behavioral despair was defined as an increase in the immobility time.

*Novel object recognition (NOR)*

Long-term memory was assessed using the NOR test[^8^](#_ENREF_8). Rats were first familiarized to the testing arena consisting of a black acrylic box (50 x 50 x 150 cm) with an open field space, for 8 min and with no objects presentation. On the following day, animals were allowed to freely explore two identical objects for 10 min. Twenty-four hours later, animals returned to the arena for 3 min, with one of the objects replaced by a novel one. The familiar and novel objects differed on size, shape, texture and color. The NOR arena was cleaned with 10% ethanol between trials to avoid odor cues. All sessions were videotaped and the time spent exploring both objects was determined manually. Analysis were conducted blind. In case of repeated testing, distinct objects were presented in each timepoint of analysis. Analysis was conducted blindly. The percentage of time spent exploring the novel object was used as a measure of long-term memory performance.

*Morris water maze (MWM) test*

To further assess cognitive function, including working and spatial reference memory as well as behavioral flexibility, the MWM paradigm was performed as previously described[^9^](#_ENREF_9). The water maze consisted in a black circular tank (diameter: 170 cm; depth: 50 cm), filled with water (23 ± 1ºC; 31 cm of depth) placed in a dimly lit room with extrinsic visual clues on the surrounding walls. The water tank was divided into four imaginary quadrants and a black escape platform (12 cm diameter; 30 cm high), invisible to rodents, was placed in the center of a quadrant. Trials were video-captured by a video-tracking system (Viewpoint, Champagne au Mont d’Or, France).

Working memory task

The working memory task was used to evaluate the cognitive domain that relies on the interplay between the hippocampal and prefrontal cortex functions[^9^](#_ENREF_9)^,^ [^10^](#_ENREF_10). An escape platform was placed in one of the quadrants and maintained during the four daily trials. The test was performed during four days, and in each day the platform was repositioned in a different quadrant. The goal of the task was to learn the position of the hidden platform and retain this information along the daily trials. For each trial, rats were placed in the water facing the wall in each of the quadrants (north, east, west or south). A trial was considered concluded when the platform was reached within the time-limit of 120 sec. The escape latency time was recorded for each trial. Performance in this task was also achieved by analyzing the area under the curve (AUC)[^11^](#_ENREF_11) and the slopes of the linear regressions of the escape latency curves (as a index of the learning capacities)[^12^](#_ENREF_12).

Spatial reference memory task

Following the working memory task (days 1-4), spatial reference memory, a hippocampal-dependent function, was assessed by maintaining the platform in the same quadrant during three consecutive days (days 4-6)[^13^](#_ENREF_13). Animals were tested in four trials, in accordance to the previously described procedure. Escape latencies and distances swam on days 4-6 were recorded and analyzed. As previously, performance in the reference memory task was determined by the AUC and the learning slope.

Behavioral flexibility task

On day 7, animals were tested in a reverse learning task (prefrontal cortex-dependent[^14^](#_ENREF_14)) by positioning the platform into a new (opposite) quadrant. Rats were tested in a four-trial paradigm, as described above, and the time-spent swimming in each quadrant was recorded. The percentage of time spent in the new and old quadrant containing the platform was used as a measure of reversal performance.

Probe

Following the behavioral flexibility task, a single probe trial was performed by removing the platform from the pool and allowing animals to search for the platform for 60 sec. The distance spent in the target quadrant was recorded.

**Immunostaining procedures**

Animals were deeply anaesthetized with sodium pentobarbital (20%; Eutasil®, Sanofi, Gentilly, France) and transcardially perfused with phosphate buffered saline (PBS) followed by cold 4% paraformaldehyde (PFA). Brains were removed, post-fixed in 4% PFA, cryoprotected in 30% sucrose overnight, and then embedded in Optimal Cutting Temperature compound (OCT, ThermoScientific, Waltham, MA, USA), snap-frozen and stored at -20ºC. Coronal sections (20 μm)

containing the hippocampal dentate gyrus (DG) were further stained to assess cell proliferation, neurogenesis and astrogliogenesis. Sections were double-stained with BrdU (#6326; rat, 1:100; Abcam, Cambridge, UK) and GFAP (#20334; rabbit, 1:200; Dako, Glostrup, Denmark) to label adult-born astrocytes or NeuN (#AB377; mouse, 1:100; Millipore, Temecula, CA, USA) to label mature newborn neurons. Neuroblasts were identified by a double-staining with Ki-67 (#AB9260; rabbit, 1:300; Millipore) and doublecortin (DCX, SC-8066; goat, 1:50; Santa Cruz Biotechnology, Dallas, TX, USA). Furthermore, double staining with Ki-67 and Sox-2 (#79351; mouse, 1:200; Abcam) was performed to detect type II progenitor cells. Cell nuclei were stained with 4',6-diamidino-2-phenylindole (DAPI, 1:200; Sigma Aldrich). The density of each cell population in the DG was determined by the ratio of the total number of double-positive cells and the respective area. Analysis and cell counting were performed using a confocal microscope (Olympus FluoViewTM FV1000, Hamburg, Germany) and an optical microscope (Olympus BX51). Observer was blinded to the experimental condition of each subject. Data are reported as number of cells *per* mm^2^.

**Neuronal morphology**

To assess the dendritic morphology of granule neurons, the Golgi-Cox staining was performed[^15^](#_ENREF_15). Animals were transcardially perfused with 0.9% saline and brains removed and immersed in a Golgi-Cox solution (1:1 solution of 5% potassium dichromate and 5% mercuric chloride diluted 4:10 with 5% potassium chromate) for 14 days. Afterwards, brains were transferred to a 30% sucrose solution and cut on a vibrotome. Coronal sections (200 μm thick) were collected in 6% sucrose and blotted dry onto gelatin-coated microscope slides, alkalinized in 18.7% ammonia, developed in Dektol (Kodak, Rochester, NY, USA), fixed in Kodak Rapid Fix, dehydrated and xylene cleared. Further three-dimensional reconstructions of neurons were performed using a motorized microscope (Olympus BX51) and the Neurolucida software (MicroBrightField Bioscience, Williston, VT, USA). Analysis of the reconstructed neurons was performed using the NeuroExplorer software (MicroBrightField Bioscience). Structural analysis included total neuronal dendritic length and 3D arrangement of neuronal branching using the Sholl analysis (number of dendritic intersections with concentric circles positioned at radial intervals of 20 µm). For each animal, at least 6 neurons were analyzed and neurons from the same animal were averaged. Observers were blinded to the experimental condition of each subject.

**RT-PCR measurements**

Oligonucleotide primers for Synapsin I (SYN1, sense CACCGACTGGGCAAAATACT, antisense TCCGAACTTCCATGTCC), Neural Cell Adhesion molecule (NCAM, sense AAAGGATGGGGAACCCATAG, antisense TAGGTGATTTTGGGCTTTGC), Doublecortin (DCX, sense CTCAGGTAACGACCAAGACGCAAAT, antisense ACTTCCAGGGCTTGTGGGTGTA), Neurogenic differentiation 1 (NEUROD1, sense ACAACAGGAAGTGGAAACATGAC, antisense ACTGAGGCACTCGTCTGTCC), Signal transducer and activator of transcription 3 (STAT3, sense TGGACCGTCTGGAAAACTGGATAAC, antisense CTCCACCACGAAGGCACTCTTCATTA), Bone morphogenetic protein 4 (BMP4, sense TCCATCACGAAGAACATCTGGAGAA, antisense GTCCACCTGCTCCCGAAATAGC), and B2M (sense GTGCTTGCCATTCAGAAAACTCC, antisense AGGTGGGTGGAACTGAGACA) were designed using Primer-BLAST software (NCBI).

**Corticosterone levels measurement**

Serum corticosterone levels in the blood serum were measured using a [^125^I] radioimmunoassay kit (MP Biomedicals, Costa Mesa, CA, USA), according to the manufacturer’s instructions. The sensitivity was 0,02 ng/mL. Sampling (tail venipuncture) was performed between 8 to 9 a.m., after the last exposure to stress. Values are expressed as the percentage of corticosterone levels relative to the control group.

**Supplementary Figures**


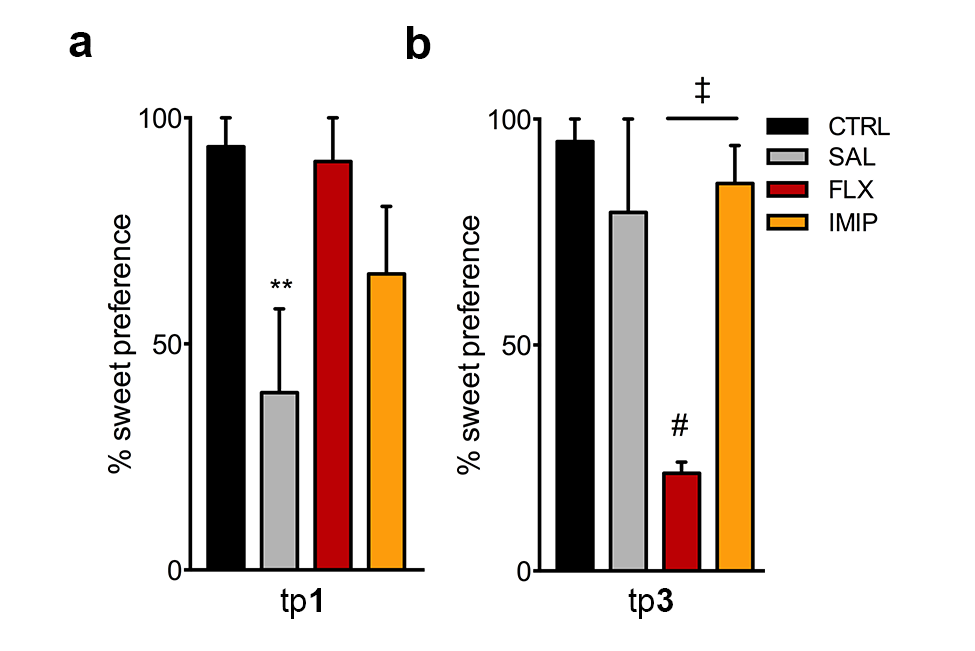


**Supplementary Figure 1.** Anhedonic-like behavior induced by recurrent stress exposures are prevented by pre-treatment with imipramine and not with fluoxetine. (**a** and **b**) Assessment of anhedonic-like behavior throughout the experimental protocol by the sweet drive test (SDT), revealed that, although both ADs are able to revert the stress-induced anhedonic-behavior (**a**), only animals treated with imipramine present no alterations, after re-exposure to stress (**b**). *Denotes the effect of uCMS analyzed by Student’s *t*-test; #Denotes the effect of ADs, by comparison of treatment and SAL animals and ‡ denotes differences between ADs, analyzed by one-way ANOVA. Data represented as mean ± SEM. ^*, #, ‡^ *P* ≤ 0.05, ^**, ##, ‡‡^ *P* ≤ 0.01; *n =* 6-8 animals *per* group. Abbreviations: TP, time-point; CTRL, non-stressed animals; SAL, animals repeatedly exposed to uCMS and non-treated animals; FLX, animals repeatedly exposed to uCMS and treated with fluoxetine; IMIP, animals repeatedly exposed to uCMS and treated with imipramine.


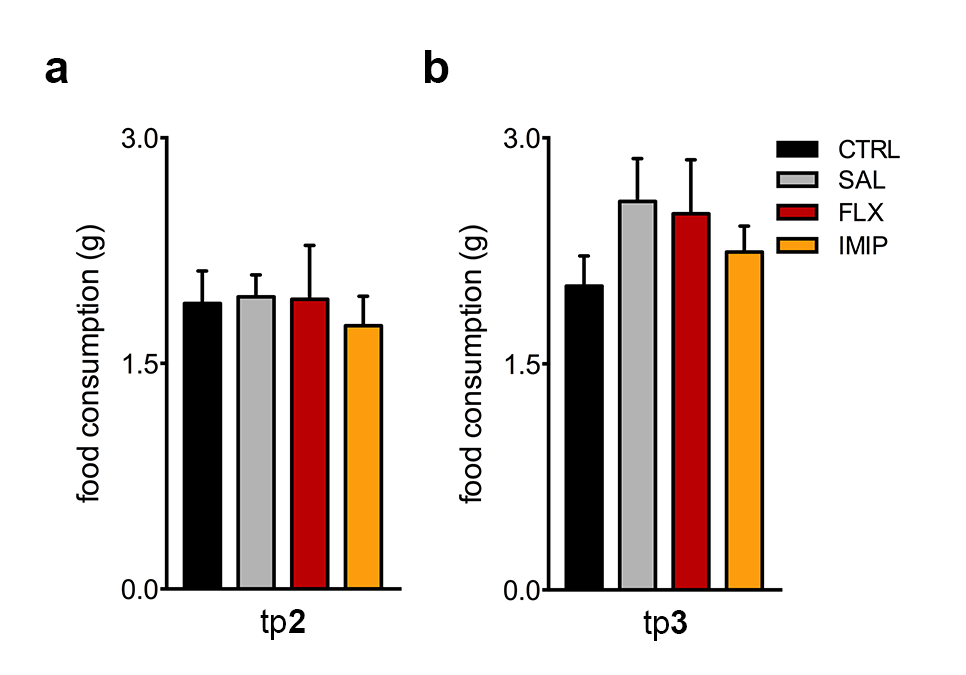


**Supplementary Figure 2.** Recurrent stress exposure had no effect on food consumption, in the NSF test. At the end of the NSF test, and for a 10-min period, food intake was assessed before (**a**, tp2) and after (**b**, tp3) re-exposure to uCMS protocol. Data represented as mean ± SEM. *n* = 6-8 animals *per* group. Abbreviations: TP, time-point; CTRL, non-stressed animals; SAL, animals repeatedly exposed to uCMS and non-treated animals; FLX, animals repeatedly exposed to uCMS and treated with fluoxetine; IMIP, animals repeatedly exposed to uCMS and treated with imipramine.


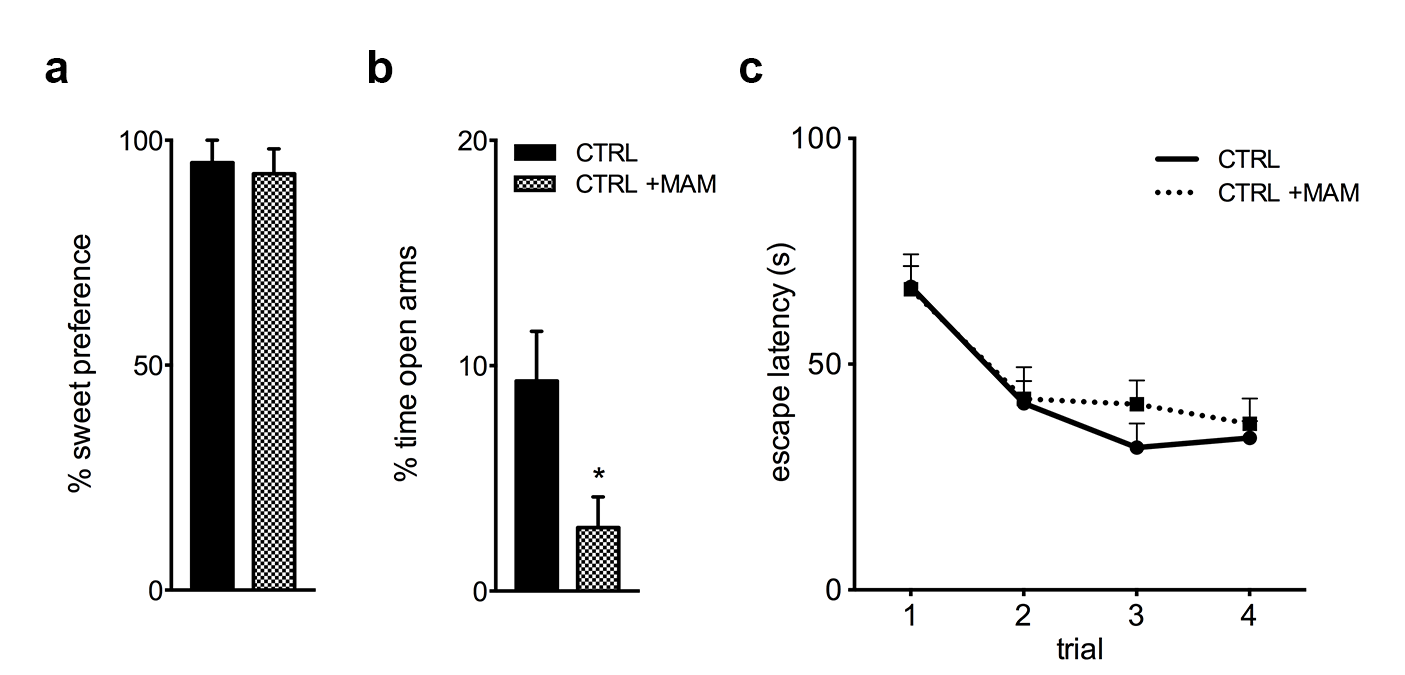


**Supplementary Figure 3.** Methyloxymethanol (MAM) negatively impacts anxiety-like behavior. Treatment of non-stressed animals with the cytostatic agent MAM do not affects anhedonic-like behavior assessed by the SDT test or working memory task in the MWM test, however induces an anxious-like state as denoted by a decrease in the % of time in the open arms of EPM test. *Denotes the effect of uCMS analyzed by Student’s *t*-test; Analysis of variance repeated measures was used to analyze cognitive learning tasks performance. Data represented as mean ± SEM. ^*^ *P* ≤ 0.05; *n* = 6-8 animals *per* group. Abbreviations: MAM, methyloxymethanol; CTRL, non-stressed animals; CTRL + MAM, non-stressed animals treated with MAM for two weeks.

**Supplementary References**

1. Bessa JM, Ferreira D, Melo I, Marques F, Cerqueira JJ, Palha JA *et al.* The mood-improving actions of antidepressants do not depend on neurogenesis but are associated with neuronal remodeling. *Mol Psychiatry* 2009; **14**(8)**:** 764-773, 739.

2. Mateus-Pinheiro A, Pinto L, Bessa JM, Morais M, Alves ND, Monteiro S *et al.* Sustained remission from depressive-like behavior depends on hippocampal neurogenesis. *Transl Psychiatry* 2013; **3:** e210.

3. Willner P. Chronic mild stress (CMS) revisited: consistency and behavioural-neurobiological concordance in the effects of CMS. *Neuropsychobiology* 2005; **52**(2)**:** 90-110.

4. Patricio P, Mateus-Pinheiro A, Irmler M, Alves ND, Machado-Santos AR, Morais M *et al.* Differential and Converging Molecular Mechanisms of Antidepressants' Action in the Hippocampal Dentate Gyrus. *Neuropsychopharmacology* 2014.

5. Mateus-Pinheiro A, Patricio P, Alves ND, Machado-Santos AR, Morais M, Bessa JM *et al.* The Sweet Drive Test: refining phenotypic characterization of anhedonic behavior in rodents. *Front Behav Neurosci* 2014; **8:** 74.

6. Walf AA, Frye CA. The use of the elevated plus maze as an assay of anxiety-related behavior in rodents. *Nat Protoc* 2007; **2**(2)**:** 322-328.

7. Porsolt RD, Bertin A, Jalfre M. Behavioral despair in mice: a primary screening test for antidepressants. *Arch Int Pharmacodyn Ther* 1977; **229**(2)**:** 327-336.

8. Vogel-Ciernia A, Wood MA. Examining object location and object recognition memory in mice. *Curr Protoc Neurosci* 2014; **69:** 8 31 31-17.

9. Cerqueira JJ, Mailliet F, Almeida OF, Jay TM, Sousa N. The prefrontal cortex as a key target of the maladaptive response to stress. *J Neurosci* 2007; **27**(11)**:** 2781-2787.

10. Kesner RP. Behavioral analysis of the contribution of the hippocampus and parietal cortex to the processing of information: interactions and dissociations. *Hippocampus* 2000; **10**(4)**:** 483-490.

11. Youngblood BD, Zhou J, Smagin GN, Ryan DH, Harris RB. Sleep deprivation by the "flower pot" technique and spatial reference memory. *Physiol Behav* 1997; **61**(2)**:** 249-256.

12. Drouin A, Bolduc V, Thorin-Trescases N, Belanger E, Fernandes P, Baraghis E *et al.* Catechin treatment improves cerebrovascular flow-mediated dilation and learning abilities in atherosclerotic mice. *Am J Physiol Heart Circ Physiol* 2011; **300**(3)**:** H1032-1043.

13. Morris R. Developments of a water-maze procedure for studying spatial learning in the rat. *J Neurosci Methods* 1984; **11**(1)**:** 47-60.

14. de Bruin JP, Sanchez-Santed F, Heinsbroek RP, Donker A, Postmes P. A behavioural analysis of rats with damage to the medial prefrontal cortex using the Morris water maze: evidence for behavioural flexibility, but not for impaired spatial navigation. *Brain Res* 1994; **652**(2)**:** 323-333.

15. Zaqout S, Kaindl AM. Golgi-Cox Staining Step by Step. *Front Neuroanat* 2016; **10:** 38.
